# Improving the Mechanical Properties of Biodegradable Polyhydroxyalkanoates via PHA–PHA Block‐Copolymer Synthesis

**DOI:** 10.1002/chem.71303

**Published:** 2026-07-09

**Authors:** Katrin Blandine Kockler, Paul Lant, Steven Pratt, Bronwyn Laycock

**Affiliations:** ^1^ School of Chemical Engineering The University of Queensland Brisbane Queensland Australia

**Keywords:** block copolymers, P3HB4HB, PHBV, polyhydroxyalkanoates

## Abstract

Polyhydroxyalkanoates (PHAs) are materials of high interest due to being bacterially synthesized and biodegradable in a variety of environments. Yet the most commonly available PHAs still lack the mechanical properties needed to truly replace a wide range of nondegradable plastics. Poly(3‐hydroxybutyrate) (P3HB) and its copolymer poly(3‐hydroxybutyrate‐*co*‐3‐hydroxyvalerate) (PHBV) exhibit high tensile strength and modulus. However, their high crystallinity leads to a lack of toughness and especially elongation, resulting in a relatively stiff and brittle material. By contrast, the copolymerization product of 3‐hydroxybutyrate (3HB) and 4‐hydroxybutyrate (4HB)—poly(3‐hydroxybutyrate‐*co*‐4‐hydroxybutyrate) (P3HB4HB)—shows a considerable improvement in elongation at break at higher 4HB contents, but lacks tensile strength and toughness, leading to a ductile but weak material. By coupling these very different PHAs together as a block copolymer, we have produced novel materials with significantly improved toughness compared to the homopolymers as well as comparable blends thereof, with an elongation at break of 630% and a toughness of 52 MJ/m^3^, more than tripling previously reported results for PHA–PHA block‐copolymers. Such attractive properties, comparable to those of commercial nondegradable thin film plastics, point to the potential for use of these biodegradable block copolymers in film applications.

## Graphical abstract

Covalent linkage of a highly crystalline PHBV with a ductile P3HB4HB is shown to produce block copolymers with markedly enhanced extensibility and toughness, outperforming equivalent blends. The results demonstrate that polymer architecture, not composition alone, governs mechanical response, offering a route to biodegradable materials that combine flexibility with toughness.

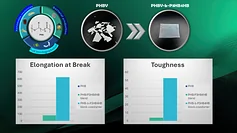

## 1 Introduction

With the consistently rising demand for plastics in our everyday lives and the increasing problems being faced worldwide regarding plastic pollution, finding environmentally friendly alternatives is a pressing issue. With research progressing into bioderived and biodegradable plastics, the requirements for such alternatives have also progressed from very minimal requirements—such as, for example, being degradable under industrial composting conditions and with limited material properties—to being biodegradable in a range of natural environments and being derived from renewable resources, all while displaying physical and mechanical properties that are competitive with common petroleum‐based commodity plastics and having drop‐in processability.

PHAs (polyhydroxyalkanoates) are a class of polyester that are naturally bioderived and fully biodegradable under ambient conditions. The microorganism‐based synthesis of PHAs from renewable feed stocks gives them an advantage over bioplastics such as polycaprolactone (PCL) or poly(butylene adipate‐*co*‐terephthalate) (PBAT), that are biodegradable but synthesized from fossil‐based raw materials like crude oil [1]. While other bioderived bioplastics are widely available, such as poly(lactic acid) (PLA) or poly(butylene succinate) (PBS), the biodegradability of these materials is limited, with degradation only being achieved in controlled facilities and industrial composting [2, 3]. PHAs on the other hand provide the benefit of full biodegradability in a range of natural environments like soil, marine, or fresh water, as well as common household composting [4]. Considering these qualifications, as well as the low toxicity and biocompatibility of PHAs, they seem to be excellent candidates for a variety of applications, including biomedical or food packaging. However, the most common and readily available PHAs, such as PHB (poly(3‐hydroxybutyrate)) and P3HB4HB (poly(3‐hydroxybutyrate‐*co*‐4‐hydroxybutyrate)), have drawbacks as standalone materials regarding their mechanical properties that considerably narrow the range of possible applications. PHB is the most common homopolymer of the PHAs and readily available in tonne scale quantities, making it an interesting material for the production of bioderived and biodegradable plastic products on an industrial scale. Yet, the high crystallinity and significant secondary crystallization post processing leads to a relatively stiff and brittle material with poor impact strength, rendering it unsuitable for certain products such as thin films [5, 6, 7], while the narrow processing window poses challenges for processability, risking degradation through β‐scission reactions at temperatures just marginally above the melting temperature [8].

One approach to modifying the physical and mechanical properties of PHB is the synthesis of random copolymers of two or more different comonomer units in the PHA backbone. These random copolymers can be microbially synthesized through the variation of feed stock and are available in a range of comonomer ratios, producing a variety of physical and mechanical properties [9, 10, 11]. Yet, not all of these biologically produced varieties are easily accessible in larger scale and many of these random copolymers still don't exhibit the desired properties.

P3HB4HB, as a random copolymer of 3HB and 4HB units, can be produced in large scale and with varying 4HB contents, with crystallinities reflecting 4HB content and ranging from semi‐crystalline to amorphous [12, 13, 14]. At higher 4HB contents (∼30 mol% and above), P3HB4HB is a naturally amorphous polymer, behaving as an elastomer at room temperature with long elongation at break. However, the increase in elasticity compared to PHB comes with a loss of tensile strength, leading to a stretchy but weak material with a low elastic modulus, unsuitable for forming durable films.

Another approach to improving final material properties is to combine two or more PHAs with different starting properties, either as a physical blend or through the formation of a block‐copolymer. While blending the two materials might be the easier and less time‐consuming approach, certain property changes can only be achieved through the covalent bonding of the starting materials via block‐copolymer formation [15, 16, 17, 18]. Employing polymer blocks that show immiscibility can lead to microphase separation within a covalently bound molecule, inducing unique microstructures and morphologies, and thus changing the mechanical behavior of the product [6, 18, 19, 20, 21].

While biological approaches to block‐copolymerization have been reported, employing strategies such as alternation of carbon feed [16, 20, 22] or the use of genetically modified organisms [16, 17, 23, 24], they display their own set of disadvantages, such as often broad product mixtures that are hard to separate, and the need for sterile and highly controlled environments. A chemical approach to block‐copolymer formation can provide a pathway that is more feasible and controlled than biological production, offering a wider variety of possible block‐combinations, while at the same time providing the means to access properties not achievable with physical blending [1, 25, 26].

While a number of different block‐copolymerization routes have been investigated based on PHAs, including copolymer candidates such as polyethylene glycol (PEG or PEO), PCL, polystyrene (PS), or polymethacrylates [27, 28, 29, 30, 31], research on the synthesis of chemically produced PHA‐*b*‐PHA and especially the investigation of their mechanical properties is still limited. A full review of the available literature can be found in Mai et al. [32].

With reference to this current work: isocyanate chemistry has been employed to produce poly(3‐hydroxybutyrate‐*co*‐4‐hydroxybutyrate)‐*block*‐poly(3‐hydroxybutyrate‐*co*‐3‐hydroxyhexanoate‐*co*‐3‐hydroxyoctanoate) (P3HB4HB‐*b*‐PHHxHO) multi‐block copolymers with molecular weights of 66–112 kDa from low molecular weight oligomers ranging from 800 to 3400 Da [33]. Similarly, multi‐block copolymers have been produced from low molecular weight oligomers of P3HB4HBs with differing 4HB contents, to produce P3HB4HB‐*b*‐P3HB4HB polyester urethanes with molecular weights of up to 81 kDa [34]. Although in both cases the prepared films are described as “stretchable and tough”, no mechanical properties are reported and—as for a majority of the PHA‐based block‐copolymers investigated—the material development was focusing more on biocompatibility and biomedical applications where high molecular weights and mechanical properties are of less importance.

Examples where mechanical properties were reported include the preparation of high molecular weight hard‐soft‐hard ABA‐type tri‐block copolymers prepared via ring opening polymerization (ROP) from various combinations of PHB, poly(3‐hydroxybutyrate‐*co*‐3‐hydroxyvalerate) (PHBV), and poly(3‐hydroxyheptanoate) (PHHp), which self‐assemble into thermoplastic elastomers [35]. The mechanical properties for these multi‐block copolymers range from tensile strength and elongation at break combinations of 25 MPa and 300% to 3 MPa and 400%, showing successful improvement of elasticity compared to PHB when combined with amorphous copolymers while, in the instance of PHB‐*b*‐PHBV‐*b*‐PHB, retaining the tensile strength.

The use of isocyanate‐based PHA‐*b*‐PHA block copolymer formation, with associated reporting of mechanical properties, has been undertaken by Andrade et al. [36], who synthesized poly(3‐hydroxybutyrate)‐*block*‐poly(3‐hydroxyoctanoate) (PHB‐*b*‐PHO) multi‐block copolymers from oligomeric low molecular weight starting materials, exhibiting relatively low final molecular weights of 10 kDa *M*_n_ and 33 kDa *M*_w_. While the elongation at break showed a moderate increase to 37% compared to PHB (at 5%), lower tensile strength and elastic modulus values (7 and 213 MPa, respectively) were consistent with its low molecular weight.

A similarity for all aforementioned PHA‐*b*‐PHA block‐copolymers is that they have been synthesized either from monomers (via ROP) or from low molecular weight oligomers forming multi‐block materials. The first examples of PHA‐*b*‐PHA block‐copolymer synthesis from comparably higher molecular weight starting materials (*M*_n_ > 20 kDa) were reported by Mai et al. [18, 37] using isocyanate chemistry to couple PHBV blocks of varying 3HV contents.

PHBV‐diols containing 1 mol% 3‐hydroxyvalerate (3HV) and 67 mol% 3HV, respectively, with number average molecular weights of 25–43 kDa, were used to form a PHBV (67% 3HV)‐*b*‐PHBV(1% 3HV)‐*b*‐PHBV(67% 3HV) tri‐block copolymer with an *M*_n_ of 100 kDa [18]. Comparison to a blend as well as a random copolymer of comparable average 3HV content showed a strong improvement in mechanical properties, with elongation at break increasing from approx. 2%–7.5% to 100% and toughness increasing from 0.1 to 15 MJ/m^3^, while also showing slight improvements in tensile strength and Young's modulus compared to the equivalent blend and random copolymer. The step‐change in mechanical properties for these block‐copolymers has been attributed to the formation of microphase domains between the highly crystalline 1 mol% 3HV and the amorphous 67 mol% 3HV domains, and the considerable increase in elongation to break and toughness shows the potential of microstructure changes and resulting mechanical improvements accessible through block formation.

While a variety of research has been published on the miscibility and properties of blends between PHBV and P3HB4HB, no work has been reported to date, to the best of our knowledge, on the formation of PHBV‐*b*‐P3HB4HB block copolymers and their associated mechanical properties, as induced by the covalent bonding of the two considerably different PHA materials. Commercially available PHA copolymers with differing comonomer contents were therefore selected, choosing copolymers that are known to be immiscible in the blend. By design, the combination of a high elastic modulus PHBV with the high elongation at break P3HB4HB is set to balance the strengths of each individual material and overcome the rigidity of PHBV, resulting in more ductile cast films. Considering both the significant improvement in elongation at break compared to pure PHBV as well as higher tensile strength and elastic modulus compared to pure P3HB4HB that have been reported in blended systems [12, 38, 39], it seems worthwhile to investigate the influence of covalently bonding the two materials in a block‐copolymer to see if a further improvement in mechanical properties compared to the blended systems can be achieved.

The present work therefore aims to investigate whether the mechanical limitations of individual PHAs can be overcome through covalent linkage rather than physical blending. Specifically, we examine the formation of block copolymers between a highly crystalline PHBV (1 mol% 3HV) and a ductile, low‐crystallinity P3HB4HB (30 mol% 4HB), and compare their thermal and mechanical properties to those of the corresponding neat materials and physical blends of comparable composition. This approach enables a systematic assessment of the extent to which covalent architecture, as opposed to composition alone, governs the resulting structure–property relationships. In particular, the study focuses on how the combination of dissimilar PHA segments influences crystallization behavior and deformation mechanisms, and whether block copolymer formation can deliver synergistic improvements in toughness beyond those achievable through blending alone.

## 2 Materials and Methods

### 2.1 Materials

PHBV random copolymer with 1 mol% 3HV content was purchased from TianAn Biopolymer (Ningbo, China) at a molecular weight of *M*_n_ = 116 kDa, *M*_w_ *= *442 kDa and a dispersity of *Ð* = 3.8. P3HB4HB with high 4HB content was purchased from CJ Biomaterials, South Korea, with a molecular weight of *M*_n_ = 166 kDa, *M*_w_ = 325 kDa and a dispersity of *Đ* = 1.95. The 4HB content has been determined via ^1^H NMR analysis to be 30 mol%, as measured by ^1^H NMR. P3HB4HB with mol 15% 4HB content was purchased from PHABuilder, China, with a molecular weight of *M*_n_ = 100 kDa, *M*_w_ = 255 kDa, and a dispersity of *Đ* = 2.6.

HPLC grade chloroform, tin(II)‐ethylhexanoate (stannous octoate, 92.5%–100%), hexamethylene diisocyanate (HDI, puriss., > 99.0% (GC)), diethyl ether (AR grade), and diethyl glycol dimethyl ether (diglyme, anhydrous) were purchased from Merck Life Science Pty Ltd and used as received. 1,2‐Dichloroethane (anhydrous 99.8%) was purchased from Merck Life Science Pty Ltd and dried over 3 Å molecular sieves for at least 24 h before use. Ethylene glycol (anhydrous, 99.8%) and dibutyltin dilaurate (95%) were purchased from Sigma–Aldrich Pty Ltd and used as received. Methanol (AR grade) was produced by ChemSupply and supplied by the UQ Science Store and used as received. Deuterated chloroform (99.8%) was purchased from NovaChem and used as received. 2‐Chloro‐4,4,5,5‐tetramethyl‐1,3,2‐dioxaphospholane (Cl‐TMDP, 95%), pyridine (anhydrous, 99.5%), cholesterol (≥99%), and chromium(III) acetylacetonate (97%) for ^31^P NMR analysis were purchased from Sigma–Aldrich and used as received.

Hydroxy‐terminated PHBV and P3HB4HB were prepared via a transesterification reaction as described in the following Section 2.2 at varying molecular weights. Blends of comparable molecular weight, as well as random copolymers with comparable 4HB content, were also analyzed to compare their properties with the produced PHBV‐*b*‐P3HB4HB block copolymer. Molecular weights of all starting materials as well as relevant blends thereof are listed in Table 1. This table also includes the data for the block copolymer product, referenced later. For Blend_90K_, values represent calculated molecular weights for a 1:1 molar physical mixture of the two components PHBV‐OH_90K_ and P3HB4HB‐30‐OH_90K_, based on independently measured GPC data of the individual components results shown in Table 1, Section 3.

**TABLE 1:** Molecular weights and composition of PHAs employed in this study.

	Material	Code	*M*_n_ [kDa]	*M*_w_ [kDa]	*Đ*	3HV [mol%]	4HB [mol%]	Comment
Raw materials	PHBV	PHBV	116	442	3.8	1	0	
	P3HB4HB	P3HB4HB	100	255	2.6	0	15	
	P3HB4HB	P3HB4HB	166	325	1.9	0	30	
Transesterification products/modified raw materials	PHBV‐OH	PHBV‐OH_40k_	41	101	2.4	1	0	Used for block‐copolymer formation
	P3HB4HB‐OH	P3HB4HB‐30‐OH_50k_	48	88	1.7	0	30	
	PHBV‐OH	PHBV‐OH_90k_	92	306	3.3	1	0	
	P3HB4HB‐OH	P3HB4HB‐30‐OH_90k_	91	152	1.7	0	30	
Blends of modified raw materials	PHBV‐OH/ P3HB4HB‐OH	Blend_50k_	48	99	2.1	0.5	15	1:1 Molar ratio
	PHBV‐OH/ P3HB4HB‐OH	Blend_90k_	92	230	2.5	0.5	15	
Block copolymer	PHBV‐*b*‐P3HB4HB	Block_90k_	80	160	2.0	0.5	15	

For clarity, three distinct material types are investigated in this study: first, the original high molecular weight starting polymers (PHBV and P3HB4HB), second, hydroxyterminated versions of these polymers prepared via glycolysis at controlled molecular weights (e.g., ∼50 and ∼90 kDa), and third, materials derived from these building blocks, comprising either physical blends of the hydroxyterminated polymers (denoted “Blend_50k_” and “Blend_90k_”) or covalently linked block copolymers formed via diisocyanate coupling (denoted “Block_80k_”). The blend nomenclature reflects the approximate molecular weight of the constituent hydroxyterminated polymers prior to mixing, rather than the molecular weight of the blend itself.

### 2.2 Synthesis of PHBV (1% HV)‐*b*‐P3HB4HB (30% 4HB) Block‐Copolymers

PHA‐*b*‐PHA block‐copolymers were synthesized using hydroxy‐terminated PHBV (1 mol% 3HV) and hydroxy‐terminated P3HB4HB (30 mol% 4HB) copolymers of varying molecular weights. Hydroxy‐terminated PHAs were produced following a previously described transesterification protocol [40]. In general, commercially available PHAs with a molecular weight of *M*_n_ = 116 kDa (PHBV) and *M*_n_ = 166 kDa (P3HB4HB), respectively, were dissolved in diglyme at 140°C under argon atmosphere. Subsequently, dibutyltin dilaurate and an excess of ethylene glycol was added, and the reaction mixture was stirred at 140°C until the desired molecular weight was obtained. In the case of both PHAs, *M_n_* of ∼90 kDa was reached after approximately 5–6 min of reaction time, while *M*_n_ of 40–50 kDa was achieved after 20 min reaction time.

Finally, the reaction was quenched by pouring into a large volume of ice–cold water and the precipitate was filtered off, washed with methanol, and dried at 80°C in vacuum.

To prepare for the block‐copolymer reaction, the glassware was dried in a vacuum oven at 100°C overnight and then connected to a Schlenk line setup and allowed to cool down under a dynamic vacuum. The solvent used was anhydrous 1,2‐dichloroethane, which was additionally dried over 3 Å mol sieves (10% *w*/*v*) for at least 24 h before use.

Hydroxy‐terminated PHAs were dried at 80°C under vacuum over night before use, then transferred into the pre‐dried 3‐neck reaction vessel fitted with a reflux condenser and rubber septum, and connected to the Schlenk line. The PHA containing vessel was then evacuated and flushed with argon gas three times. After the last evacuation/flushing cycle of the solid PHA starting materials, the reaction setup was kept under a constant argon flow and dried 1,2‐dichloroethane was added via syringe through the septum to a PHA concentration of up to 200 mg/mL. The reaction mixture was then heated to 100°C and stirred under reflux until full dissolution of the PHAs.

Subsequently, the solution was allowed to cool down to 65°C and stannous octoate (0.8 µL/mL solvent) and hexamethylene diisocyanate (HDI, 2.2 mol eq NCO:OH) were added to the reaction mixture. The solution was stirred under argon flux at 65°C for 6 h. Previous experiments have shown that after 6 h, the majority of the reaction has taken place and further reaction time increases the risk of unwanted side products forming (Figure 1) [37, 41, 42].

**FIGURE 1:**
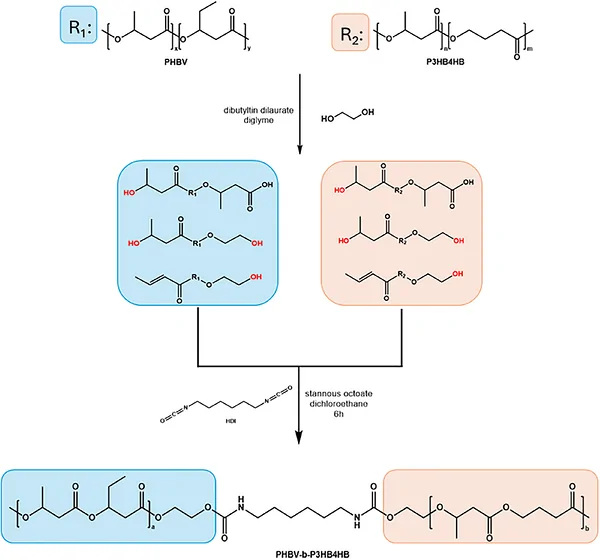
Graphical overview of glycolysis reaction of the two starting materials (PHBV and P3HB4HB) and diisocyanate‐based block‐copolymer formation. The structures shown represent segments of high molecular weight polymers; repeat units are depicted schematically for clarity.

The reaction was quenched by precipitating into a cold mixture of diethyl ether:methanol 20:1 *v*/*v* and the precipitate was filtered, rinsed with methanol, and dried at 80°C in vacuum before analysis.

### 2.3 Preparation of Films for Mechanical Testing

Thin films for mechanical testing were prepared via solvent casting into flat glass petri dishes. For the preparation of solvent cast films, 1 g of polymer sample was dissolved in 12 mL of HPLC grade chloroform at 80°C (∼5 wt%) until well dissolved. For blends of the starting block materials, 1 g of overall sample was dissolved, comprised of a 1:1 molar ratio of both copolymers. After dissolution, the sample was allowed to cool down to room temperature and filtered through a fine pore metal mesh into a glass petri dish. The dish was covered with a glass cover and aluminum foil and the solvent slowly evaporated in the fume hood over the course of several days. The resulting film was dried under vacuum at room temperature overnight and left in the open environment for two weeks before testing.

### 2.4 Material Characterization

#### 2.4.1 Nuclear Magnetic Resonance Spectroscopy (NMR)

^1^H NMR spectra were acquired at 298 K in deuterated chloroform (CDCl3) (10 mg/mL) on Bruker Advance 500 spectrometers. The number of scans was set to 512. The relative peak intensities of ^1^H‐NMR spectra were determined using MestReNova software. Chemical shifts were referenced to the residual proton peak of CDCl_3_ at 7.26 ppm. ^31^P‐NMR was performed after phosphorylation of the samples to facilitate measurement of the hydroxyl (─OH) and carboxyl (─COOH) end groups. 50 mg of sample was dissolved in 1 mL of anhydrous CDCl_3_. 100 µL of a standard solution of cholesterol (0.1 M in anhydrous CDCl_3_:pyridine 1:1.6 solution) containing chromium(III) acetylacetonate as relaxation agent was added, resulting in 0.01 mmol cholesterol standard hydroxyl group per 50 mg PHA. Afterward, 50 µL of 2‑chloro‐4,4,5,5‐tetramethyl‐1,3,2‐dioxaphospholane (Cl‐TMDP) was added. The mixture was allowed to react on a shaker for a minimum of 2 h. After phosphorylation of the end groups, the sample was tested by ^31^P‐NMR with a scan number of 1024. The resulting PHA hydroxyl peak was compared to the integral of the internal cholesterol standard.

#### 2.4.2 Gel Permeation Chromatography (GPC)

The number‐average molar mass (*M*_n_), weight‐average molar mass (*M*_w_), and dispersity (*Đ*) of the PHA copolymers were determined by GPC, using an Agilent 1260 Infinity II Multidetector GPC System. Samples were dissolved in HPLC grade chloroform (∼3 mg/mL) followed by filtration using polytetrafluoroethylene syringe filters (0.22 µm, Kinesis, ESF‐PT‐13–022). A column set consisting of a guard column (Agilent PLgel (10 µm, 7.5 mm × 50 mm)) followed by 3 × Agilent PLgel MIXED‐B (10 µm, 7.5 mm × 300 mm) columns in series were used. The columns were kept at 30°C. A refractometer, at 30°C, was used to detect the signals. A chloroform flow rate of 1 mL/min was used for the analysis. The apparatus was calibrated with narrowly distributed molecular weight PS standards and PS‐H EasiVial calibration standards (PL2010‐0201). The Mark–Houwink–Sakurada (MHS) relation and specific MHS parameters (*K* and *α*) were used to correct the molar mass. The following Mark–Houwink constants for PHB in chloroform, *K* = 7.7 × 10^−3^ mL/g and *α* = 0.82, were used in this study.

#### 2.4.3 Differential Scanning Calorimetry (DSC)

DSC analysis was performed on a TA instrument Q2000 under nitrogen flow (50 mL/min) with sample masses of 2–4 mg in sealed aluminum pans. A multi‐step heating/cooling profile was employed with the following steps: First, equilibrate at 25°C followed by heating to 185°C with a 10°C/min ramp, keeping isothermal at 185°C for 0.2 min; Second, Cooling from 185°C to −70°C with a 10°C/min ramp, keeping isothermal at −70°C for 5 min; Third, heating from −70°C to 185°C with a 10°C ramp; Fourth, cooling from 185°C to −70°C with a 40°C/min ramp; Fifth, heating to 80°C with a 20°C/min ramp. The first heating cycle was used to determine melting temperatures *T*_m_ and melting enthalpy *ΔH*_m_, while the first cooling cycle was used to determine the crystallization temperatures *T*_c_. Glass transition temperatures *T*_g_ were determined from the final heating cycle. Data analysis was performed using TA Universal Analysis software.

For these materials, crystallinity determination by DSC relies on the assumption of a well‐defined enthalpy of fusion for a fully crystalline reference phase. While such values are well established for PHB, with a thermodynamic Δ*H*° of 146 J/g [43], there is no universally accepted thermodynamic Δ*H*° for P4HB or P(3HBco4HB), and reported values are empirical and composition dependent. In addition, in the present system (blends and block copolymers), overlapping or weak melting contributions from the 4HBrich phase, together with phase separation and constrained crystallization, prevent reliable deconvolution of individual phase contributions. As a result, calculation of an absolute crystallinity for the total material is inherently uncertain. Accordingly, we are using melting enthalpy (*ΔH*_m_) directly and interpreting this as a relative measure of crystalline content, rather than presenting absolute crystallinity values.

#### 2.4.4 Mechanical Property Assessment

Tensile testing of the block‐copolymer as well as starting materials and blends was performed according to the ISO 527 standard under ambient conditions using a universal testing machine Instron 5543 quipped with a 50 N load cell and pneumatic grips. Samples were razor cut using a stainless‐steel template into rectangular shape with average film thickness of 0.1–0.15 mm measured via Tecloc gauge, according to ASTM D882. Sample dimensions were 5 mm width and 40 mm sample length, with a 20 mm grip distance.

Tensile testing of each polymer sample was repeated on three to four specimens, with the exception of Blend_50K_ for which only two samples gave reportable results. Samples were measured at a grip separation speed of 2 mm/min until fracture. The tensile modulus was determined from the slope of initial low strain in the elastic regime of curves, and the tensile toughness was calculated as an integrated area under the curve.

#### 2.4.5 Statistical Method for Processing of Data

All data were processed in GraphPad Prism. For datasets with *n* ≥ 3, error bars represent standard deviation. For datasets with *n* = 2, individual data points are shown, and no statistical dispersion is reported.

## 3 Results and Discussion

Previous investigations into the reaction progression of diisocyanate coupling reactions showed that the reaction seems to plateau after approximately 6 h with no further increase in molecular weight during longer reaction periods. However, extended reaction times increase the risk of unwanted side‐products through branching and crosslinking of the diisocyanate linker [41, 42]. Therefore, as a general guideline, the isocyanate reactions for the formation of block‐copolymers were terminated after a maximum of 6 h reaction time.

The molecular weights for all materials including reaction products are given in Table 1.

The block‐copolymer *M*_n_ and *M*_w_ values are approximately double those of the starting materials, indicating that mostly di‐block copolymers have been formed. Although the presence of PHBV and P3HB4HB “homopolymers” cannot be excluded—either from unreacted material or from coupling of PHBV with PHBV and P3HB4HB with P3HB4HB chains, respectively—the differences in mechanical properties compared to blends of the starting materials support the assumption that the majority of the polymers formed consists of A–B type PHBV‐*b*‐P3HB4HB block‐copolymer. Since the dispersity of the block‐copolymer products remained relatively low and unchanged (Figure 2), a wide mixture of multi‐blocks is not expected. In addition, an excess of diisocyanate can lead to the formation of allophanate groups, resulting in side‐products and crosslinking of the polymer chains. However, such products generally form insoluble gels and the allophanate groups can be detected with NMR spectroscopy. No gelatinous or insoluble components were observed during the workup and filtration of the product and no allophanate groups where visible in the product NMR spectra, indicating that no significant by‐product formation or crosslinking has occurred.

**FIGURE 2:**
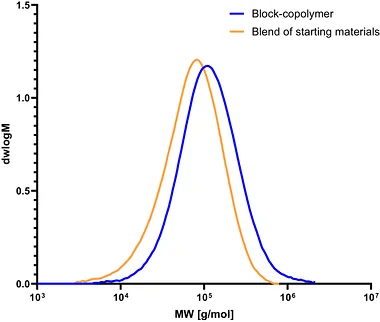
GPC traces of a blend of the starting materials (Blend50k, made from 1:1 ratio of PHBV‐OH40k and P3HB4HB‐OH50k, orange line) and the resulting block‐copolymer (Block80k, PHBV‐b‐P3HB4HB, blue line). Corresponding molecular weight data is collated in Table 1.

### 3.1 Thermal Properties

To determine changes in thermal behavior between starting materials, a blend thereof, and the formed block‐copolymer, the thermal properties of the diisocyanate coupling products were compared to the raw starting materials (*M*_n_ = 41 kDa for PHBV and *M*_n_ = 48 kDa for P3HB4HB), a PHBV/P3HB4HB blend of comparable molecular weight (*M*_n_ = 90 kDa, molar ratio PHBV and P3HB4HB of 1:1), and a random copolymer P3HB4HB‐15 with15% 4HB content and a molecular weight of *M*_n_ = 100 kDa (Table 2). Original DSC thermograms can be found in the Supporting Information.

**TABLE 2:** Thermal properties of polymer starting materials, blends, and block‐copolymer product.

	Material	Code	*T*_m_ [°C]	*ΔH*_m_ [J/g]	*T*_c_ [°C]	*ΔH*_c_ [J/g]	*T*_g_ [°C]
Materials as received from supplier	PHBV	PHBV	172.4	99.2	116.7	92.9	—
	P3HB4HB	P3HB4HB‐30	83.8	2.5	49.8	0.41	−16.6
	P3HB4HB	P3HB4HB‐15	171.0	12.5	79.5	27.4	−10.6
Starting materials after glycolysis	PHBV‐OH	PHBV‐OH_90k_	173.2	95.9	117.5	83.5	—
	P3HB4HB‐OH	P3HB4HB‐30‐OH_90k_	81.6	0.24	61.3	0.16	−19.5
	PHBV‐OH	PHBV‐OH_50k_	172.2	74.0	105.9	74.5	—
	P3HB4HB‐OH	P3HB4HB‐30‐OH_50k_	86.1	0.9	—	—	−20.1
PHA blends ratio 1:1	PHBV‐OH/ P3HB4HB‐OH	Blend_90k_	172.7	47.3	117.5	37.82	−18.45
	PHBV‐OH/ P3HB4HB‐OH	Blend_50k_	171.8	61.5	114.2	65.2	−17.8
Block‐copolymer	PHBV‐*b*‐P3HB4HB	Block_80k_	169.2	36.8	103.2	31.1	−18.8

While pure P3HB4HB‐30 (30% 4HB content) shows a minor melting peak at considerably lower temperatures than pure PHBV (at 83°C vs. 173°C), with almost negligible melting enthalpy of 2.5 J/g (vs. 95.4 J/g for PHBV), blending the materials did not lead to a significant decrease in *T*_m_, with both blends (Blend_90k_ and Blend_50k_) sitting at around 171°C. The block‐copolymer formed between PHBV‐OH_40k_ and P3HB4HB‐30‐OH_50k_ also showed only minor decrease in melting temperature, to *T*_m_ *_= _* 169°C. However, the decrease in melting enthalpies from pure PHBV to blend to block‐copolymer (99.2 J/g (PHBV); 47.3 J/g (PHBV/P3HB4HB Blend_90k_); 36.8 J/g (PHBV‐b‐P3HB4HB block)) indicates a decrease in crystallinity with the introduction of 4HB into the mixture and additional decrease during block‐copolymer formation. There are a number of possible influences from the addition of P3HB4HB in both blends and blocks. First, if the crystallization rate of the PHBV phase remains unchanged, the addition of amorphous P3HB4HB acts as a dilution of the crystalline component, lowering the overall crystallinity of the material. Second, the addition of amorphous P3HB4HB results in the formation of micro‐ and macro‐phase separated domains in both blends and blocks, constricting chain migration and inhibiting the crystallization by reducing the crystallization ability of PHBV in the mixed materials [39]. This is particularly evident for blocks, where the covalent linkages tying the blocks together limits the possibility of complete chain migration into a macrophase separated domain, so that the effects on thermal properties are more pronounced when P3HB4HB and PHBV are covalently linked in a block‐copolymer rather than simply combined in a physical blend. When comparing the block‐copolymer to the random copolymer P3HB4HB‐15 (15 mol% 4HB) containing a comparable 3HB:4HB ratio, the melting enthalpy of the block‐copolymer sits between those of the blend and the P3HB4HB‐15, indicating a lower crystallinity for the random copolymer, while the *T*_g_ of the random copolymer is considerably higher than both blend and block. Since the 4HB units act as crystal defects in the copolymer and therefore disrupts the crystallinity of the PHB [14], it seems reasonable that the random copolymer exhibits considerably smaller crystalline areas, regularly disrupted by 4HB units, compared to the block‐copolymer where the 4HB‐induced disruption is more localized on one (P3HB4HB) block.

For all samples containing P3HB4HB, a clear *T*_g_ has been observed. The blends as well as block‐copolymer have exhibited a single *T*_g_, which could normally be attributed to miscibility of the two materials. However, neither of the pure PHBV samples showed a clearly definable *T*_g_, due to the very high crystallinity of PHBV and hence the small proportion of amorphous phase—which is the phase that displays the glass transition property. Therefore, the lack of two distinct *T*_g_s in blends and block‐copolymer does not necessarily allow to draw conclusions about miscibility of the employed materials.

### 3.2 Mechanical Properties

To the best of our knowledge, there is no research published on block‐copolymers of PHBV with P3HB4HB. However, there are several publications investigating the properties and miscibility of PHBV/P3HB4HB blends [38, 39]. While these publications describe blends containing P3HB4HB of differing 4HB contents, a finding they have in common is that the addition of amorphous or semi‐crystalline P3HB4HB increases the elongation at break while at the same time reducing the elastic modulus.

When Wang et al. blended a very high molecular weight (*M*_n_ = 1.3 MDa) P3HB4HB with 19 mol% 4HB content with PHBV (1 mol% HV, *M*_n_ = 250 kDa) at a 50:50 ratio—resulting in a ∼10 mol% overall 4HB content in the blend—the elongation at break increased from 2.3% to 356%, with a slight loss of tensile strength and reduction of elastic modulus by approximately 50%. Interestingly, when increasing the P3HB4HB content to 80% in the blend, they recorded a slightly increased tensile strength again, closer to the reported tensile strength of the pure P3HB4HB (23 MPa), with a further significant loss of elastic modulus [40].

Luo et al. on the other hand investigated a range of blends of PHB with a 41 mol% 4HB P3HB4HB and found significantly lower values for both tensile strength and elongation. Comparing their blends to the pure PHB, improvement in elongation was observed for all blend ratios, increasing from 1.8% for pure PHB to 27% for a 50:50 blend (∼20 mol% 4HB content in blend), with a similar high loss of elastic modulus from 1.3 GPa to 177 MPa. However, they also observed a significantly higher loss in tensile strength from 15.2 to 2.3 MPa [39]. In this case, no data was reported on the pure 41 mol% 4HB P3HB4HB, as they stated they were not able to cast any stable films of the pure materials due to their general weakness.

The P3HB4HB random copolymer employed in this study for the synthesis of block‐copolymers lies approximately between these two examples, with a 4HB content of ∼30 mol%. The issues encountered in this study with casting stable films of the pure 30 mol% 4HB P3HB4HB material were similar to those reported by Luo et al. [39] and successful casts showed an extremely low tensile strength of only 0.5 MPa, in stark contrast to the values reported by Wang et al. [40]. The elongation at break for blends, however, was considerably higher than those reported by Luo [39]. This shows that the literature values reported here are to be considered cautiously when wanting to compare results, as differences in sample preparation, analytical instruments, measurement methods and data analysis might make a direct comparison challenging. Therefore, in the following discussion about the mechanical properties of the materials produced in this study, direct comparison will only be drawn to starting materials and blends that have been measured in the course of this study using consistent methods and conditions.

In contrast to PHA‐*b*‐PHA studies using low molecular weight oligomers as starting materials to form multi‐block polymers, this work is based on the formation of di‐block copolymers with a single urethane linker in the main chain. Previous investigations have shown that at this low concentration (<0.1 wt%) the urethane linker has no influence on the mechanical properties of the product [38].

The mechanical properties of the as‐produced block‐copolymers are compared to the as‐purchased starting materials PHBV (1 mol% HV, *M*_n_ = 116 kDa) and P3HB4HB (30 mol% 4HB, *M*_n_ = 166 kDa), the as‐purchased random copolymer P3HB4HB with comparable 4HB content (15 mol% 4HB, *M*_n_ = 100 kDa), as well as to blends of the hydroxy functionalized building blocks with comparable molecular weights, in Figure 3. Original stress‐strain curves can be found in the Supporting Information.

**FIGURE 3:**
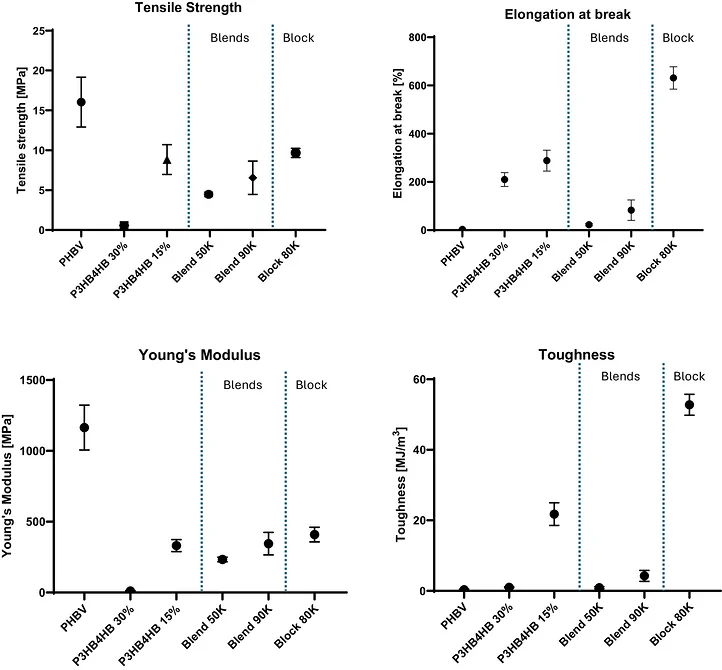
Comparison of tensile strength, elongation at break, elastic modulus, and toughness of the tested materials. Materials displayed on the *x*‐axis are, from left to right: pure PHBV (PHBV—1 mol% HV, *M*_n_ ∼100 kDa), P3HB4HB (P3HB4HB30%—30 mol% 4HB, *M*_n_ ∼170 kDa and P3HB4HB15%—15 mol% 4HB, *M*_n_ ∼100 kDa), a blend of the starting materials used for block‐copolymer formation (Blend 50K—50:50 molar ratio: PHBV—1 mol% HV, *M*_n_ ∼50 kDa and P3HB4HB—30 mol% 4HB, *M*_n_ ∼50 kDa), a blend of starting materials with comparable molecular weight to the block copolymer product (Blend 90K—50:50 ratio: PHBV—1 mol% HV, *M*_n_ ∼90 kDa and P3HB4HB—30 mol% 4HB, *M*_n_ ∼90 kDa), and the block‐copolymer product (Block 80k—50:50 ratio PHBV 1 mol% HV—P3HB4HB 30 mol% 4HB, overall *M*_n_ ∼80 kDa). Data are presented as mean ± standard deviation, with *n* = 3 for PHBV, P3HB4HB 30%, P3HB4HB 15%, and Block 80K, and *n* = 4 for Blend 90K, except for Blend 50K where *n* = 2, wherein only individual data points are shown.

When comparing the mechanical properties of the 50/50 blends of polymers of different molecular weights—where Blend_50k_ uses the same materials as employed for the block‐copolymer formation (PHBV‐OH_50k_ and P3HB4HB‐30‐OH_50k_) and Blend_90k_ uses polymeric materials that each have a similar molecular weight to the resulting block‐copolymer (PHBV‐OH_90k_ and P3HB4HB‐30‐OH_90k_)—it can be seen that the molecular weights of the PHAs have some influence on the mechanical properties of the resulting blend, indicated by an increase in elongation at break and toughness of the material when going from 50 to 90 kDa molecular weight of the blend. Although almost doubling the molecular weight from 50 to 90 kDa, both blends are still in a relatively low molecular weight range and the change in mechanical properties is not particularly large. However, when investigating the block‐copolymer of a similar molecular weight (*M*_n_ = 80 kDa), the changes in mechanical properties between blend and block become very obvious. The most drastic change can be observed in the elongation at break, which on average stayed below 85% for both blends, while for the block‐copolymer it exceeded 600% elongation.

A similarly dramatic increase is shown in the overall toughness of the material, at greater than 50 MJ/m^3^ for the block‐copolymer compared to values below 5 MJ/m^3^ for the 30 mol% 4HB P3HB4HB and PHBV starting materials as well as both blends. While the elongation at break for the pure P3HB4HB‐30 is reported in Figure 3 to be around 200%, the actual elongation might be considerably higher, yet the difficulties in casting a stable film made mechanical testing on the material challenging. However, even considerably higher elongations would most likely keep the toughness of the material below 5 MJ/m^3^ due to the extremely low tensile strength of the pure P3HB4HB‐30.

In agreement with the previously mentioned studies on PHBV/P3HB4HB blends, all samples containing any content of 4HB showed significant reduction in their elastic modulus, from 1.3 GPa for pure PHBV to 230–410 MPa for blends and block‐copolymer, although the block‐copolymer still shows a slightly elevated modulus compared to the blends.

A comparison of the PHBV‐*b*‐P3HB4HB block‐copolymer to a random P3HB4HB copolymer containing a comparable overall 4HB content of approximately 15 mol% was also of interest. The mechanical data for P3HB4HB‐15 was measured on the as‐supplied material, with an *M_n_* of ∼100 kDa, which is an only slightly higher molecular weight than for the synthesized block‐copolymer, suggesting that an influence of the molecular weight on the mechanical properties is not expected. However, direct comparison of the two materials (Block_80k_ vs. P3HB4HB‐15) shows obvious differences, especially in elongation at break and toughness of the material. While the measured tensile strengths are approximately the same, the elongation at break for the block‐copolymer was roughly double that of the random copolymer P3HB4HB‐15, leading to a similar increase in overall toughness of the material from ∼20 to 50 MJ/m^3^. While P3HB4HB‐15 exhibited a lower elastic modulus than the blend, the block‐copolymer with the same 4HB content shows the highest modulus of the 4HB containing samples, at 410 MPa.

Previous works have attributed the step‐change in mechanical properties mostly to microphase separation in the block‐copolymer system, with the two (or more) materials of the blocks being separated in crystalline/amorphous or hard/soft segments that are immiscible. However, miscibility studies on PHBV/P3HB4HB blends suggest that phase separation is not a given when considering these two materials. When Wang et al. [40] investigated PHBV/P3HB4HB blends (19 mol% 4HB), they reported no evidence of phase separation across the composition range studied. In contrast, Luo et al. [39] observed a transition from miscibility to immiscibility at higher 4HB contents, with phase separation emerging above ∼50 wt% P3HB4HB, corresponding to overall 4HB contents exceeding ∼20 mol%. Consistent with this, Ishida et al. [12] have generally described immiscibility between 3HB‐rich and 4HB‐rich materials.

Since the work presented in this study covers an approximately 50:50 mixture of PHBV with a 30 mol% 4HB P3HB4HB—leading to an overall 4HB content of ∼15 mol%—it might be worthwhile to consider if the material might still be within the threshold of miscibility, not phase separating. In this context, the mechanical behavior cannot be interpreted solely in terms of a simple crystalline–amorphous balance. While the low melting enthalpy associated with the P3HB4HB component reflects a reduced contribution from ordered structures under the conditions investigated, the resulting mechanical response is also governed by how these regions are distributed and interact within the material. In the absence of clear evidence for full phase separation at these compositions, the increase in ductility is therefore attributed not only to reduced overall crystallization, but also to changes in the connectivity between more and less ordered regions. In the block copolymer, this connectivity is imposed by covalent linkage between segments, modifying how deformation is accommodated compared to the corresponding blend and enabling large increases in elongation and toughness without requiring fully phase‐separated domains.

This would make it of particular interest to see if additional significant changes in mechanical properties could be achieved by further increasing the 4HB content, either through employing a P3HB4HB block with a higher molar percentage of 4HB or by increasing the length of the P3HB4HB block to push the overall 4HB content of the block‐copolymer to higher values.

## 4 Conclusion and Outlook

A successful formation of a PHA–PHA block‐copolymer comprised of an approximately 1:1 mixture by mass of PHBV (1 mol% HV) and P3HB4HB (30 mol% 4HB) has been achieved via diisocyanate based coupling reaction, as evidenced by an increase in the molecular weight of the material and presence of the urethane groups in ^1^H‐NMR spectra. Further indication of successful block‐copolymer formation is given by the considerable change in mechanical properties compared to the employed starting materials as well as blends thereof. To the best of our knowledge, this work describes the first reported synthesis and analysis of a covalently bound PHA–PHA block‐copolymer based on PHBV and P3HB4HB. Comparison with blends of the employed starting materials as well as blends of PHBV and P3HB4HB of comparable molecular weights to the synthesized block‐copolymer has shown improvement in the mechanical properties, especially in elongation at break (630%) and toughness (52 MJ/m^3^), leading to a considerably less brittle and more versatile material and demonstrating the significant changes that can be achieved by covalently bonding two materials compared to a physical blend. When comparing the block‐copolymer to a random copolymer with an approximately equal amount of overall 4HB content (P3HB4HB‐15) it becomes evident that the mechanical and thermal properties are not only governed by the overall 4HB content, but also by the microstructure of the material, as indicated by significant changes in elongation, toughness, and overall crystallinity.

While the results presented in this paper already show a significant improvement of material properties, there are some interesting questions yet to be answered for the possibility of further material improvement. While the molecular weights of all materials introduced in this study are above the critical entanglement molecular weight (typically ∼20 kDa), at which polymers exhibit a transition to entangled behavior and associated changes in viscosity and elasticity, it is well established that further increases in molecular weight, above 100,000–150,000 Da molecular weight, can lead to continued improvements in mechanical properties, particularly toughness and elongation at break [44, 45, 46].

For the block‐copolymer presented in this study, the *M*_n_ sits just below 100 kDa; crossing into higher molecular weight regions could lead to another significant change in viscosity with a possible influence on the mechanical properties.

Furthermore, studies of PHB/P3HB4HB blends have suggested a critical 4HB content in the blend of approximately 20 mol%, above which the materials become immiscible. While in a blend this immiscibility of the blended materials and resulting phase separation might not be desirable, in block‐copolymers previous studies have shown that a microphase separation can induce step‐changes in mechanical properties. The block‐copolymers investigated in this study have an overall 4HB content of approximately 15 mol%, leaving an interesting opportunity to answer the question if increasing the 4HB content in the block‐copolymer—either through a higher mol%4HB P3HB4HB block or different PHBV:P3HB4HB ratio—would bring on further improvement of material properties due to an onset of internal immiscibility of the covalently bound blocks and resulting microphase separation.

Last, while the synthesis of PHA–PHA block‐copolymers has shown a substantial improvement in elongation at break and toughness of the material, its robustness for certain processing methods, such as film blowing, is yet to be determined. More work is required to establish impact on processing window and thermal stability as well as whether or not the material strain hardens, as required for film blowing.

## Conflicts of Interest

The authors declare no conflicts of interest.

## Supporting information




**Supplementary File 1**: chem71303‐sup‐0001‐SuppMat.docx

## Data Availability

The data that support the findings of this study are available from the corresponding author upon reasonable request.
