# Supplementary material for: Improving the Mechanical Properties of Biodegradable Polyhydroxyalkanoates via PHA–PHA Block‐Copolymer Synthesis
Source: Chemistry. 2026 Jul 9;32(39):e71303. doi: 10.1002/chem.71303 (PMC13715456; doi:10.1002/chem.71303)
Supplement: Supplementary file 1 [file CHEM-32-e71303-s001.docx]

**Supporting information**

**Improving the mechanical properties of biodegradable polyhydroxyalkanoates via PHA-PHA block-copolymer synthesis**

Katrin Blandine Kockler^a^, Paul Lant^a^, Steven Pratt^a^, Bronwyn Laycock^a^*

*^a^The University of Queensland: School of Chemical Engineering, Brisbane, Queensland, 4072, Australia*

*E-mail address: [b.laycock@uq.edu.au](mailto:b.laycock@uq.edu.au)

**S1. DSC thermograms of PHA materials**

Figure S 1. First DSC heating scans. Left: PHBV-OH and P3HB4HB-OH starting materials, and a blend thereof. Right: PHBV/P3HB4HB blend, P3HB4HB random copolymer with 15% 4HB content, and PHBV-b-P3HB4HB block-copolymer.

Figure S 2. First DSC cooling scans. Left: PHBV-OH and P3HB4HB-OH starting materials, and a blend thereof. Right: PHBV/P3HB4HB blend, P3HB4HB random copolymer with 15% 4HB content, and PHBV-b-P3HB4HB block-copolymer.

Figure S 3. Second DSC heating scans. Left: PHBV-OH and P3HB4HB-OH starting materials, and a blend thereof. Right: PHBV/P3HB4HB blend, P3HB4HB random copolymer with 15% 4HB content, and PHBV-b-P3HB4HB block-copolymer.

Figure S 4. Second DSC cooling scans. Left: PHBV-OH and P3HB4HB-OH starting materials, and a blend thereof. Right: PHBV/P3HB4HB blend, P3HB4HB random copolymer with 15% 4HB content, and PHBV-b-P3HB4HB block-copolymer.

Figure S 5. Final DSC heating scans. Left: PHBV-OH and P3HB4HB-OH starting materials, and a blend thereof. Right: PHBV/P3HB4HB blend, P3HB4HB random copolymer with 15% 4HB content, and PHBV-b-P3HB4HB block-copolymer.

**S2. Stress-Strain curves of tested PHA materials**

Figure S 6. Stress-strain curve of PHBV-OH starting material used for blends and block-copolymer synthesis.

Figure S 7. Stress-strain curve of P3HB4HB-OH starting material containing 30% 4HB, used for blends and block-copolymer synthesis.

Figure S 8. Stress-strain curve of P3HB4HB random copolymer containing 15% 4HB, used for comparison purposes.

Figure S 9. Stress-strain curve of PHBV/P3HB4HB blend with M_n_=50kDa used for comparison with starting materials and block-copolymer.

Figure S 10. Stress-strain curve of PHBV/P3HB4HB blend with M_n_=90kDa used for comparison with block-copolymer.

Figure S 11. Stress-strain curve of the PHBV-b-P3HB4HB block-copolymer synthesised in the course of this study.
